# Methods to Determine and Analyze the Cellular Spatial Distribution Extracted From Multiplex Immunofluorescence Data to Understand the Tumor Microenvironment

**DOI:** 10.3389/fmolb.2021.668340

**Published:** 2021-06-14

**Authors:** Edwin Roger Parra

**Affiliations:** Department of Translational Molecular Pathology, The University of Texas MD Anderson Cancer Center, Houston, TX, United States

**Keywords:** multiplex immunofluorescence, matrix construction, cellular spatial distribution, nearest neighbor, correlation functions

## Abstract

Image analysis using multiplex immunofluorescence (mIF) to detect different proteins in a single tissue section has revolutionized immunohistochemical methods in recent years. With mIF, individual cell phenotypes, as well as different cell subpopulations and even rare cell populations, can be identified with extraordinary fidelity according to the expression of antibodies in an mIF panel. This technology therefore has an important role in translational oncology studies and probably will be incorporated in the clinic. The expression of different biomarkers of interest can be examined at the tissue or individual cell level using mIF, providing information about cell phenotypes, distribution of cells, and cell biological processes in tumor samples. At present, the main challenge in spatial analysis is choosing the most appropriate method for extracting meaningful information about cell distribution from mIF images for analysis. Thus, knowing how the spatial interaction between cells in the tumor encodes clinical information is important. Exploratory analysis of the location of the cell phenotypes using point patterns of distribution is used to calculate metrics summarizing the distances at which cells are processed and the interpretation of those distances. Various methods can be used to analyze cellular distribution in an mIF image, and several mathematical functions can be applied to identify the most elemental relationships between the spatial analysis of cells in the image and established patterns of cellular distribution in tumor samples. The aim of this review is to describe the characteristics of mIF image analysis at different levels, including spatial distribution of cell populations and cellular distribution patterns, that can increase understanding of the tumor microenvironment.

## Introduction

Multiplex immunofluorescence (mIF) facilitates detection of cell phenotypes (Parra et al., 2020) and quantification of spatial relationships among cells within the tumor microenvironment (Barua et al., 2018). Studying the spatial distribution of tumor cells and infiltrating immune cells in tumor samples using data obtained via mIF-based digital image analysis allows for detailed characterization of cell-cell associations and the geographic distribution of cell phenotypes, which may help in predicting clinical responses and mechanisms of resistance of cancer to immunotherapies (Yu et al., 2020). With increases in the volume and complexity of this type of data, integration of computational analysis with image analysis has become more important and relevant to better understanding the tumor microenvironment. Analysis of spatial data requires specific tools and techniques to look at these data from different angles. Over the past few years, my group has applied computational analysis tools in an exploratory way to measure the intensity of expression of cell phenotypes in cancer and the spatial distribution of cells in images obtained using mIF (Barua et al., 2018). We have also applied careful inferential methods to validate the results of cell distance analysis. In essence, we attempted to extract features from many mIF images and captured the most relevant features that can answer our questions. Once these features are extracted and checked for anomalies, hypothesis tests and mathematical models can be designed to assess the effect of certain features or patterns of cell distribution on cancer (Robinson et al., 2020). This analysis of spatial cell distribution can be used to determine whether a strong association exists between cell distribution patterns and clinicopathologic information or outcome.

Feature extraction from mIF digital image analysis begins with computing maps for individual markers using the center of the cells, which then creates a point process object. A point process from the image analysis is a collection of points that can be structured using two-dimensional coordinates in the *x*- and *y*-planes using identified cell markers (Parra et al., 2020). Creating this point process object allows us to superimpose point patterns of different markers for combined co-localization analysis, which identifies specific cell phenotypes that correspond to a unique image identifier, and each image has a corresponding case. Lastly, each cell has a binary entry for each marker that the cell expresses. This enables efficient assignment of a phenotype to each cell.

When we explore image analysis data, the cell phenotype frequencies on each mIF digital image must be counted to determine the number of pairwise phenotype incidences. We count the interaction of protein markers in every cell in the data and organize by image and case. For each cell phenotype, we estimate the intensity of another phenotype by counting the cells in a neighborhood and also increasing the radius (Illian et al., 2008). This measure of intensity is very important when adjusting for the effect of other features and computing the space between cells. Using the coordinates that the images provide after image analysis, for any image and cell phenotype, we can calculate the distance to every other cell in the image. Thus, we can construct a distance matrix that encodes the distances for all pairs of cells, giving us the opportunity to map cell pathways in every image (Illian et al., 2008). The spatial distribution of the cell phenotypes can be used to calculate several characteristics of the cells using a mathematical function that is most appropriate for the research question. Using the data provided by this method, we can model features of cellular spatial distribution to determine whether certain phenotypes differ in their patterns of distribution. For instance, we can study patterns of distribution of and distances between cells across images and cases and correlate this information with clinical data to see if the spatial distribution of these cells plays an important role in driving different responses to treatments and outcomes in the tumor microenvironment.

Herein, I describe strategies and mathematical models and functions used to study the spatial distribution of cell phenotypes in tumor tissues, demonstrating a practical approach to study the tumor microenvironment. I also discuss the integration of these analyses with their biological interpretation to answer research questions.

## Spatial Cellular Distribution

The tumor microenvironment is a complicated machinery that includes several groups of cells, such as epithelial and endothelial cells and a large variety of infiltrating immune cells, including cells involved in both the innate and adaptive immune responses to the tumor. The location and organization of these different immune cell phenotypes have emerged as important pieces of information for determining the function of these cells across tumor compartments and recognizing the possible impact of the cells on clinical outcomes in cancer patients (Masugi et al., 2019). Knowing the location of different cell populations in a tumor and the spatial distribution of the cells with other cell groups allows us to characterize a tumor to predict its response to treatment and the potential for progression and relapse. The spatial distribution of different cell phenotypes is known to be important in characterizing the tumor microenvironment, which influences recruitment of immune cells, and the microenvironment can be characterized in different regions within a tumor or studied to determine whether specific cell phenotypes are present (Tsujikawa et al., 2020). Therefore, data obtained from mIF-based digital image analysis are particularly useful for calculating functional spatial distribution metrics.

### Geographic Cell Distribution in Tumors

**FIGURE 1:**
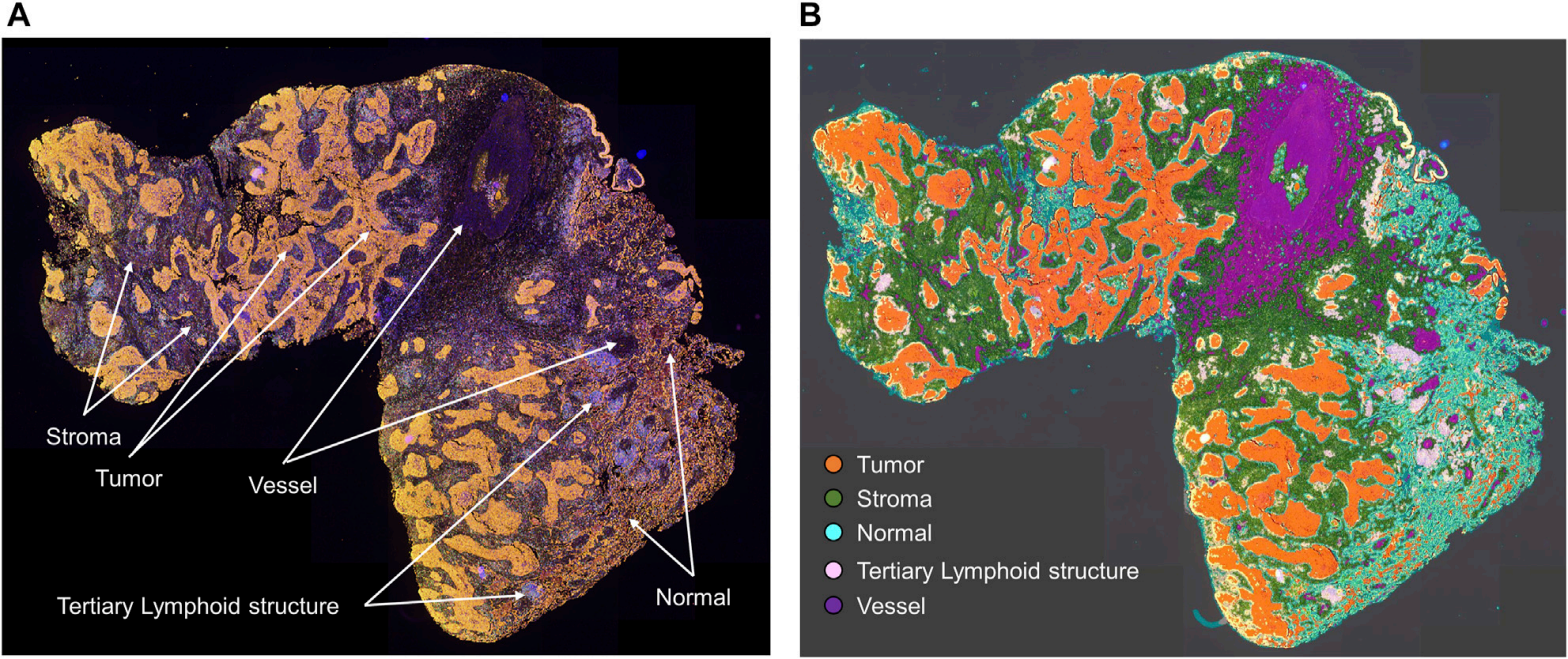
Microphotographs of a representative whole tumor section of lung squamous cell carcinoma obtained using multiplex immunofluorescence staining. **(A)** Geographic distribution of cells in different tissue compartments. **(B)** The geographic compartments were delineated using HALO image analysis software (Indica Labs, Albuquerque, NM) to analyze cells in different compartments. Original magnification, ×4.

As shown in Figure 1, studying different cell phenotypes according to their distribution in tumors, such as in the tumor and stromal compartments, normal tissue and tertiary lymphoid structures, vessels, or tumor periphery, can provide important information about the specific role of that cell phenotype (Bremnes et al., 2011; Dieu-Nosjean et al., 2014), and cellular distribution can be associated with outcomes in various tumor types. For example, T-cell populations in the tumor compartment, but not in the stromal compartment, are associated with favorable prognoses in colorectal cancer (Galon et al., 2006; Nazemalhosseini-Mojarad et al., 2019), ovarian cancer (Zhang et al., 2003), urothelial carcinoma (Wang et al., 2015), head and neck squamous cell carcinoma (Zhou et al., 2019), esophageal adenocarcinomas (Stein et al., 2017), triple-negative breast cancer (Sugie et al., 2020), pancreatic ductal adenocarcinoma (Masugi et al., 2019), and non-small cell lung carcinoma (Parra et al., 2016; Tuminello et al., 2019). Research has also shown that cytotoxic T-cells in the tumoral compartment are potential negative prognostic factors in invasive breast cancer (Catacchio et al., 2019). Furthermore, larger populations of specific cell phenotypes, such as FOXP3+ T-regulatory cells, in the tumoral compartment than in the peripheral compartment can correlate with aggressive tumor behavior, as observed with some papillary thyroid cancers (French et al., 2010). The distribution of T-cell phenotype populations across different geographic compartments can have therapeutic implications (Cooper et al., 2016; Feldmeyer et al., 2016; Parra et al., 2018) and drive the improvement and discovery of new treatments based on T-cell tumor tissue distribution.

### Spatial Distribution at the Single-Cell Level

**FIGURE 2:**
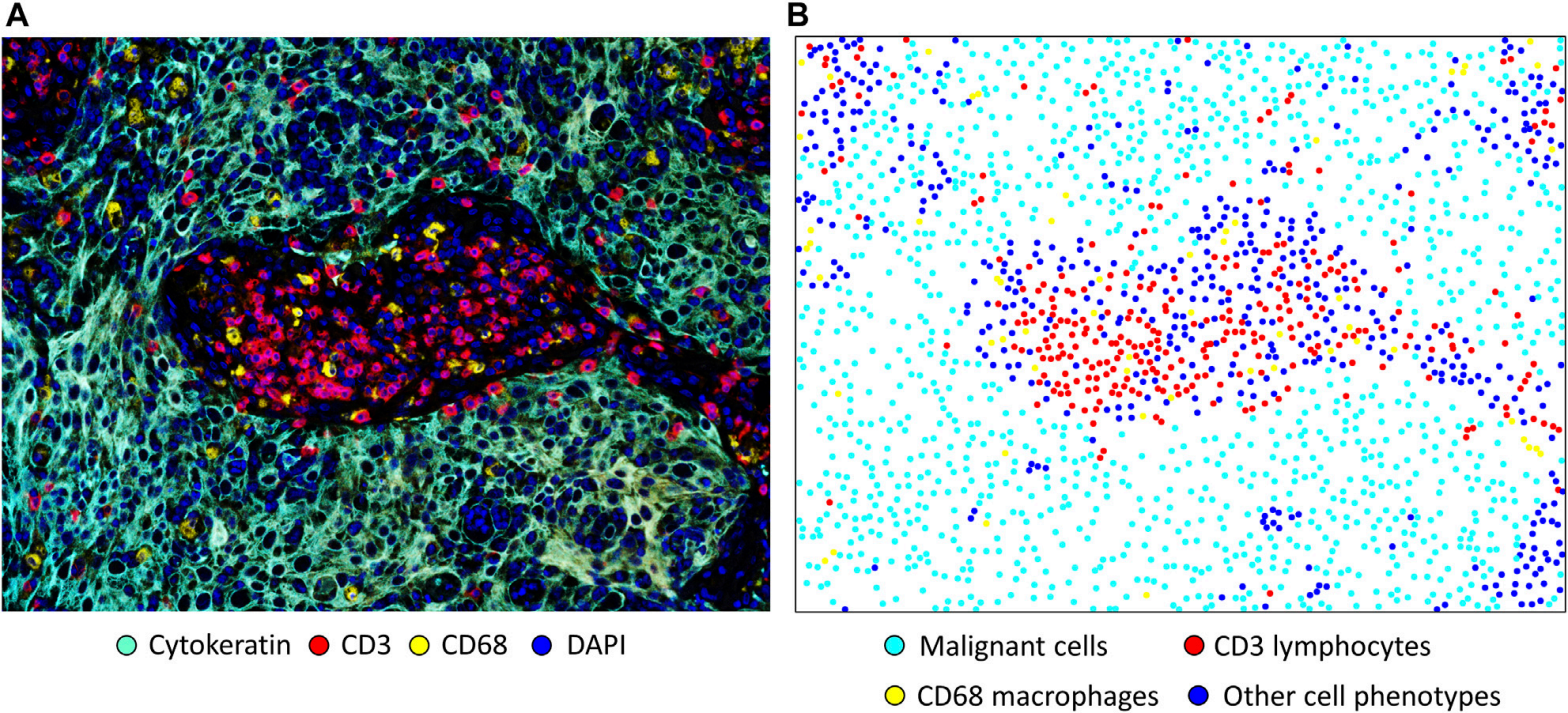
Microphotographs of a representative section of lung squamous cell carcinoma obtained using multiplex immunofluorescence staining with a panel containing cytokeratin, CD3^+^ T-cells, CD68 ^+^ macrophages, and DAPI. **(A)** A region of interest with basic marker staining. **(B)** Colored dots representing the individual cells shown in A, showing the location of each cell in the image. Original magnification, ×20, from VectraPolaris scanner and processed by Inform software (Akoya Biosciences).

In spatial cellular image analysis, images show a collection of various cell phenotypes that are identified by staining for a combination of markers in an mIF panel (Figure 2A), and these markers are translated as colored dots with x and y coordinates (Figure 2B). This analysis is not limited to single images but rather uses groups of images that are related to several tumor samples in a study. In our analysis, we consider the point pattern from our mIF image a non-parametric process, which assumes a stationary or homogeneous point pattern configuration independent of a specific location. Although only small or a few areas of observation can be considered non-stationary processes showing only a few groups of phenotypes, these areas, given the heterogenicity of the sample across images, ultimately generate dynamic ecologic patterns that may influence tumor progression and response to treatment (Gentles et al., 2015). Furthermore, study of spatial cell distribution has demonstrated its relationship with outcomes in cancer patients. For example, in non-small lung cancer, the proximity of macrophages to malignant cells was inversely correlated with prognosis; those with tumors in which macrophages were close to malignant cells had worse outcomes than those with tumors in which macrophages were far from the malignant cells (Zheng et al., 2020). Similarly, in a gastric cancer study, the proximity of FOXP3+ T-regulatory cells to CD8^+^ cytotoxic T-cells was inversely correlated with prognosis (Wang et al., 2020).

## Functional Spatial Distribution Metrics

The existing methods used in spatial analysis are many and varied. Researchers have ample opportunity to explore different techniques of cellular spatial analysis for tumor tissues and implement them using mathematical models to extract mIF image data.

In spatial image analysis, consideration of intensity and density is needed. Intensity is the absolute number of cells or their abundance in an image when looking directly into it, and density is the number of cells per unit area (cells/mm^2^).

**FIGURE 3:**
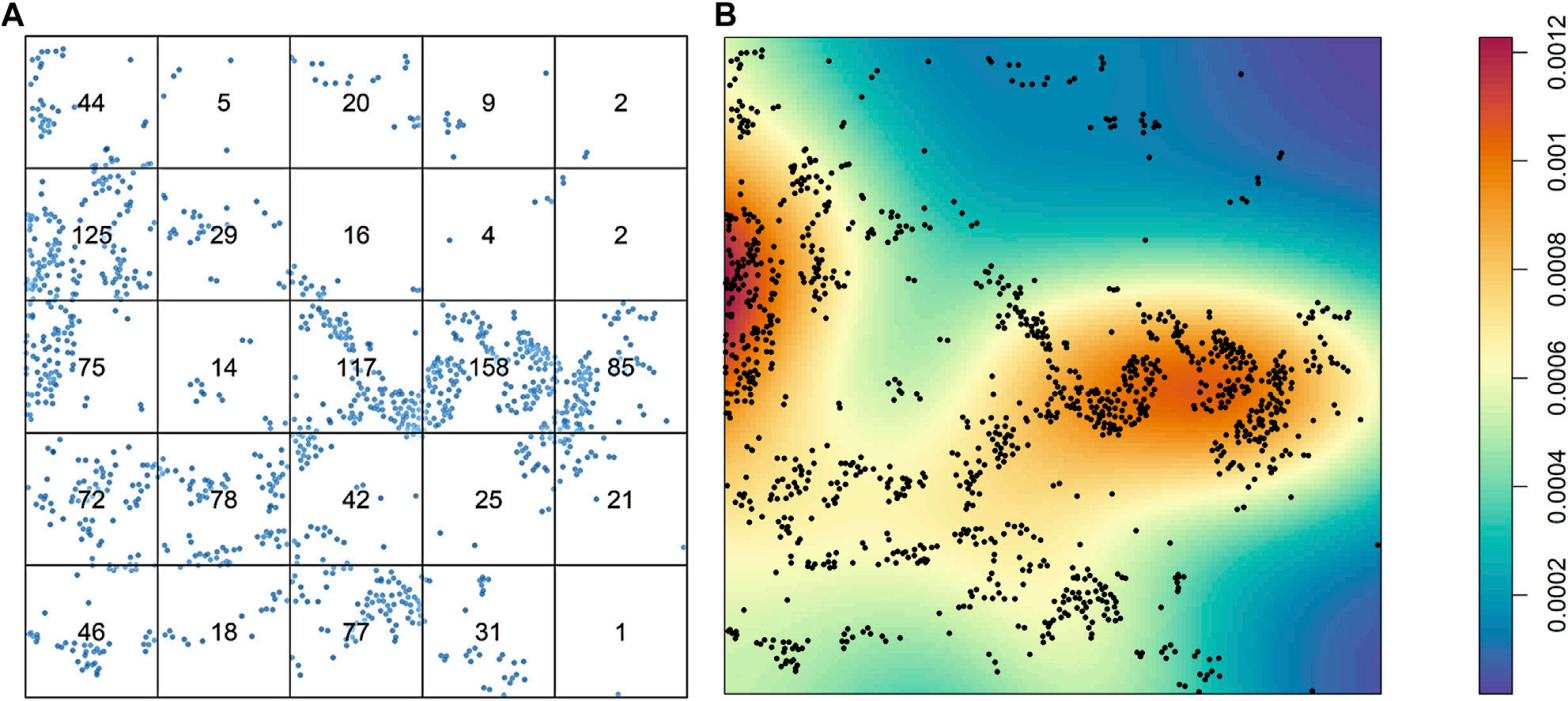
Characterization of the distribution of cells from a sample of lung adenocarcinoma in a multiplex immunofluorescence image. **(A)** Results of quadrant analysis, with each quadrant containing the exact number of cells in a square, represented by dots. **(B)** Heat map of Kernel smoothing from the same area of the image, showing areas of high and low proliferation of cells, identified by colored regions (see color scale at right). The images were generated using R studio software version 3.6.0.

After intensity and density are defined, the distribution of the cells overall is the first aspect in an image that can be studied. The cells can be distributed homogeneously or not, and a simple way to consider this variable is to divide the images into quadrants of equal size and count the cells in each quadrant. Naturally, if the number of cells varies greatly among the quadrants, the distribution of the cells is not homogeneous (Figure 3A). The distribution of cells in an image is very unlikely to be homogeneous, and overall, a good assumption is that patterns of cells will never be homogeneous. One obvious drawback to this approach to analyzing the distribution of cells across an image is the dependence on quadrant size or application of other geometric shapes of the partitions. If the quantification or application of the quadrants is not done carefully, no useful information will be drawn. Nonparametric approaches, such as kernel smoothing (Baddeley et al., 2015), are other popular methods of graphically determining whether cellular distribution is homogeneous, and these methods are useful for observing cell proliferation patterns or hot spots in an image (Figure 3B).

## Spatial Descriptive Functions

In studying the spatial relationships among different cells and their patterns of distribution in an image, several spatial descriptive functions can be applied. Basically, two groups of mathematical or computational functions can be used to analyze the data obtained in digital image analysis. One group is used to describe the measured distances between cell populations; this group includes the G-function, F-function, and J-function. The other group is used to describe the relative intensity of the cells in terms of distance measured, and this group includes the K-function, L-function, and pair correlation function. Similar principles are used to construct both function groups (Baddeley et al., 2015; Illian et al., 2008), and because these functions examine the relationship between two cell populations (*i*-to-*j*), all functions are cross-functional or mark-independent.

**FIGURE 4:**
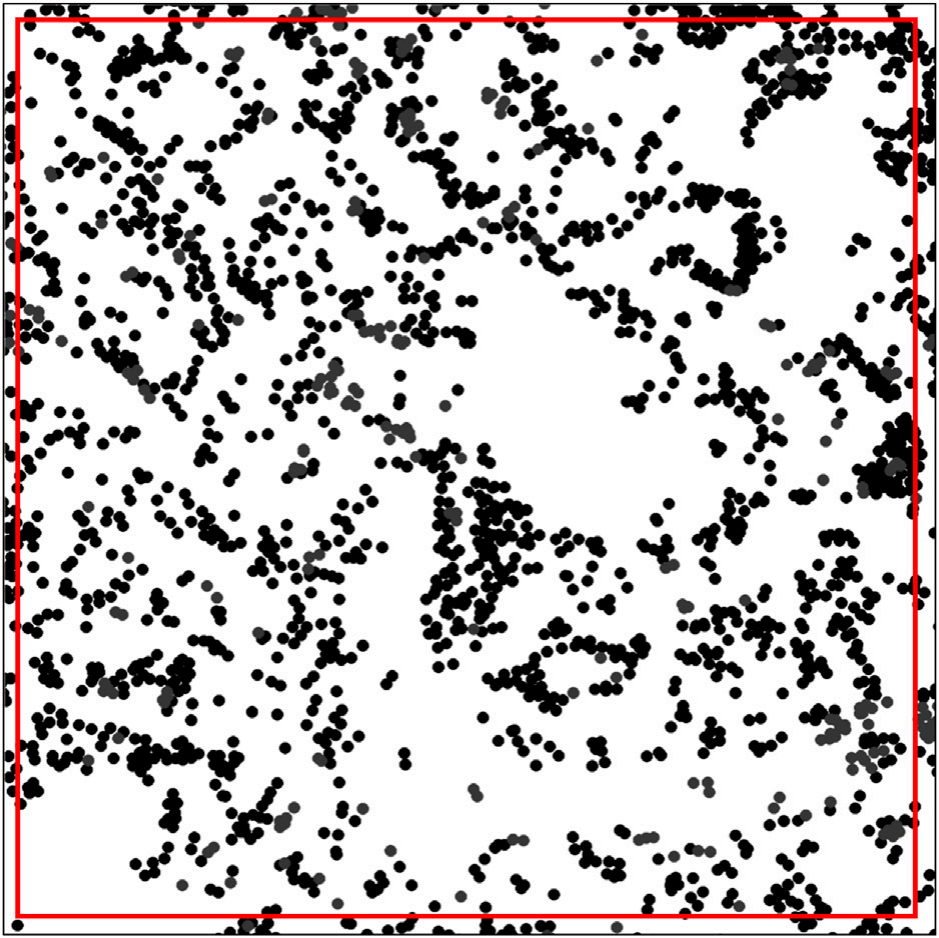
Distribution of dots representing the cell distribution patterns in an image with edge correction, showing a square marked by a red line constructed using spatstats software in the R computing language. Only the dots inside the red line were considered in the analysis of the spatial distribution pattern. The image was generated using R studio software version 3.6.0.

To apply these functions to spatial image analysis data from mIF images, users are encouraged to employ the well-known spatstats package in the R computing language (Baddeley and Turner, 2005) because it has correction tools such as edge correction, which are important for any spatial image analysis (Figure 4).

## Distance Matrix

**FIGURE 5:**
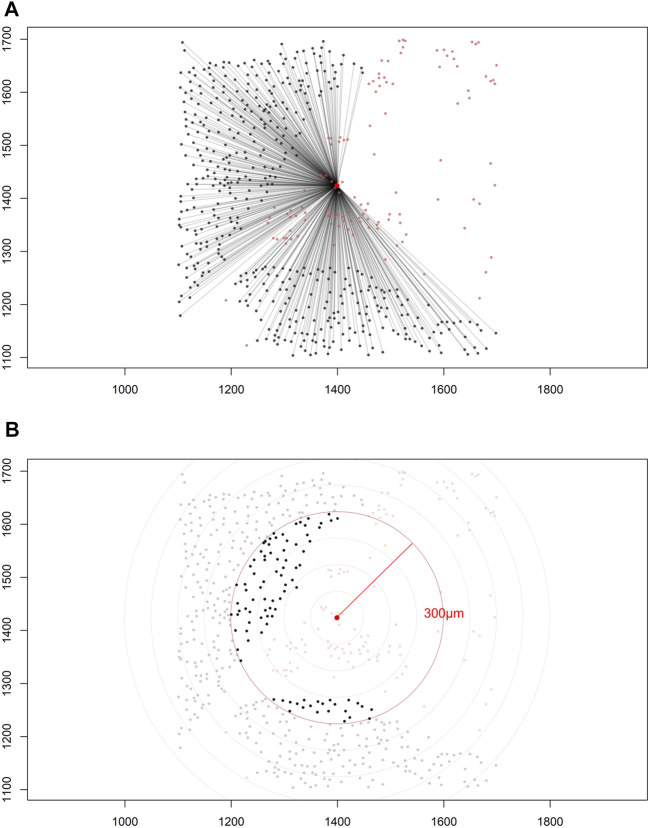
Distance matrices. **(A)** Identified cell coordinates and distance measurements from one cell phenotype (red dots) to another cell phenotype (black dots) in a lung adenocarcinoma image. **(B)** The intensity of one cell phenotype (black dots) was calculated at a given radius (red circle, 300 μm) from the other cell phenotype (red dots). The images were generated using R studio software version 3.6.0.

Construction of a distance matrix is the first step in developing any tool to reveal spatial properties of cells in an image. To maintain the simplicity of the analysis, we can assume that distances between cells are always measured in a two-dimensional Euclidean space on images that are flat. Only the cell coordinates are needed to build a distance matrix; this allows extraction of spatial information regarding the interaction between two distinct types of cells by applying various mathematical formulas on the matrix itself (Figure 5A).

**FIGURE 6:**
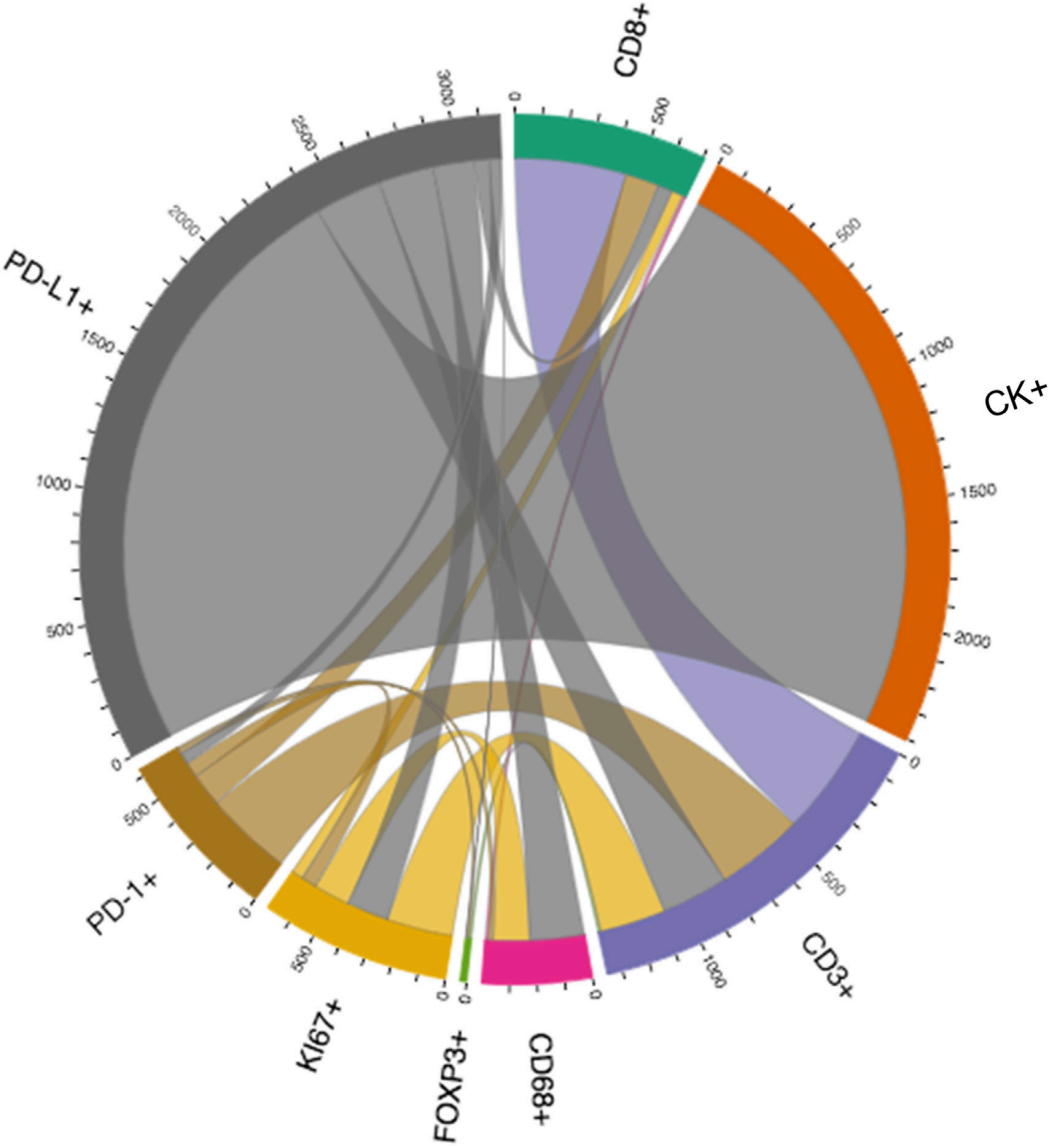
Graphic representation of a distance matrix using a chord diagram showing the flows or connections between the markers included in a multiplex immunofluorescence panel. The chord diagram shows various connections between markers that generate cell phenotypes from a multiplex immunofluorescence panel; these markers include cytokeratin (CK), CD3, CD8, FOXP3, PD-1, PD-L1, KI67, and CD68. The graphic was generated using R studio software version 3.6.1.

Depending on the specific formula applied, various features of the spatial interaction between cells can be studied. In constructing a distance matrix, the coordinates of the cell phenotypes are first ordered in rows and columns, where the rows in the matrix correspond to the number of cells from one specified cell phenotype and the columns correspond to the number of cells from another specified cell phenotype. A good visual representation of the connection between cell markers in the matrix can be obtained using a chord diagram (Figure 6).

**FIGURE 7:**
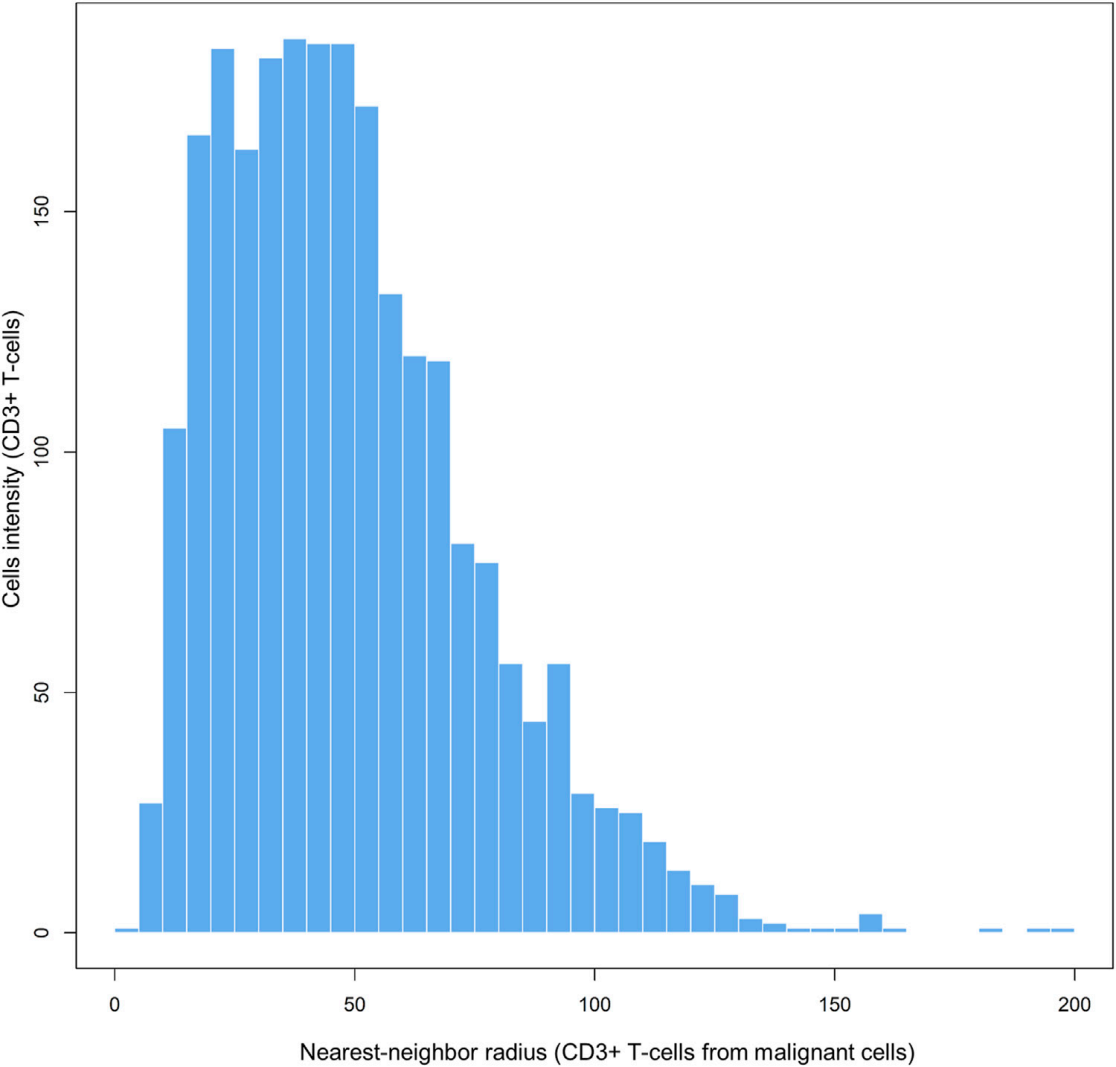
Bar graph showing the distribution of CD3^+^ T-cell distances from malignant cells across different radii, from representative data extracted from a lung adenocarcinoma sample. The graphic was generated using R studio software version 3.6.1.

Each matrix entry is the distance between one cell phenotype and another cell phenotype; in this way, all entries between two groups of cell phenotypes are displayed in the distance matrix. As mentioned above, the distance is measured for every pair of cells, i.e., from one cell phenotype of interest to another cell phenotype of interest, or, in a more simplistic way, from point A (*i*) to point B (*j*) in a given radius (*r*; Figure 5B). The maximum distance between two cells is the farthest distance between A and B in the image; this distance is limited by the region of interest analyzed. A meaningful measure must be constructed by determining the distance between each entry in column (*i*) from one cell phenotype and each entry in row (*j*) from the other cell phenotype, for example malignant cells and CD3^+^ T-cells (Figure 7). This is important when constructing other metrics for other cell phenotypes to observe the distribution of cells and to obtain a vector of distances from each cell phenotype to its nearest neighbor of another cell phenotype.

## Nearest Neighbor

**FIGURE 8:**
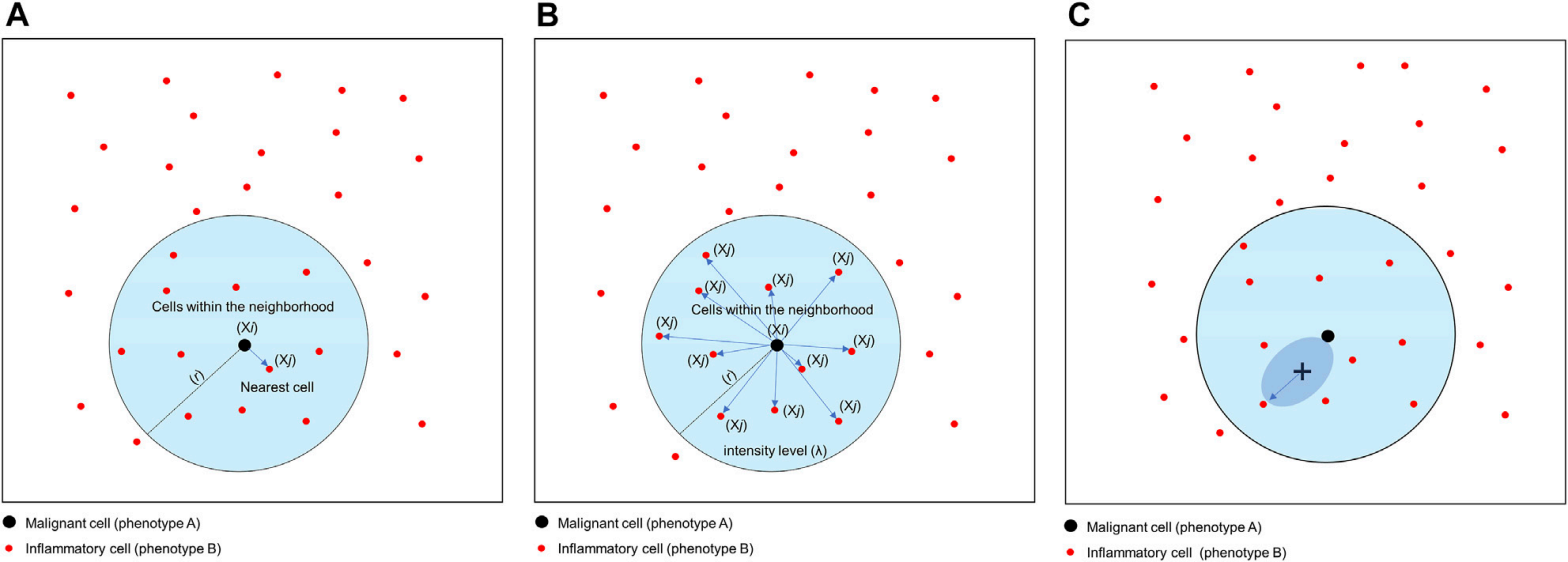
Graphics of nearest neighbor distance, K-function and empty-space F-function. **(A)** Distance matrix showing the concept of nearest neighbor distance, used to determine the probability of encountering a cell of a specific phenotype [X*i*, inflammatory cell (phenotype B)] within a certain radius (*r*) centered on another cell phenotype [X*j*, malignant cell (cell phenotype A)]. **(B)** Distance matrix showing the concept of the K-function to determine the intensity level (*λ*) of cells of a certain phenotype within a certain radius (*r*) of another cell phenotype. In this example, the function is calculating the *λ* of cell phenotype B (X*j*) from cell phenotype A (X*i*) in a point pattern. **(C)** Graphic representation of the empty-space F-function, which compares distances from an arbitrary point (x) to the nearest neighbor cell or point. The graphics were generated using R studio software version 3.6.1.

The nearest neighbor distance is used to determine the probability (*P*) of encountering a cell (point, *X*) of a specific phenotype (*j*; e.g., cell phenotype B, CD3^+^) within a certain radius (*r*) centered on another cell phenotype (*i*; e.g., cell phenotype A, malignant cells; Figure 8A) (Barua et al., 2018). This approach allows you to determine the minimum distance between each cell of phenotype A and the nearest neighbor cell of phenotype B. Of note, this distance will be completely different if measured in the opposite direction (from cell phenotype B to cell phenotype A). The direction to be evaluated (from cell phenotype A to B or vice versa) depends on the research question and is based on biological knowledge of the tumor. For instance, a researcher may wish to measure the distance from malignant cells to the nearest neighbor T lymphocytes in a certain radius, assuming that the T lymphocytes are there because of the malignant cells.

**FIGURE 9:**
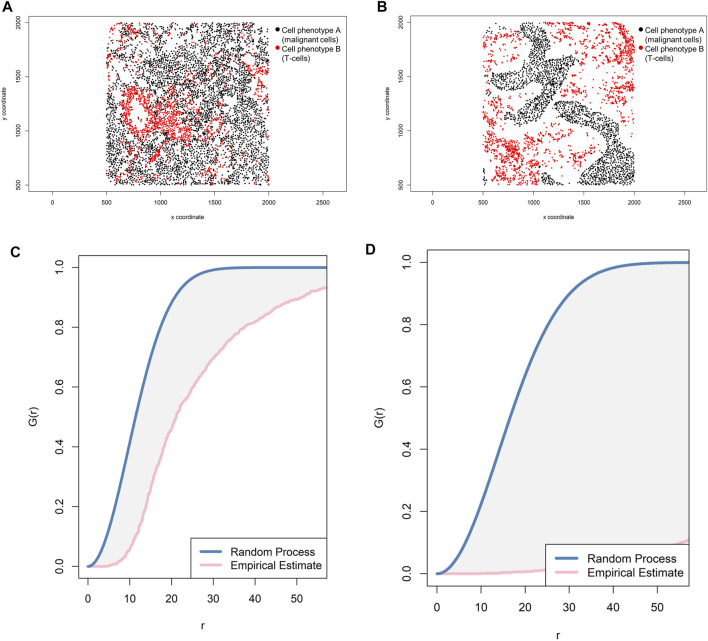
Representative graphs showing the G-function [G(*r*)] curve of point pattern distributions for cell phenotype A (malignant cells) to cell phenotype B (T-cells) in a representative multiplex immunofluorescence image of lung adenocarcinoma. **(A)** Random point pattern distribution of cell phenotype A and B. **(C)** Graphic representation of the image shown in A using the cross-G-function, showing the proximity of the G(*r*) curve to the theoretical estimate curve (Poisson curve), characterizing a random or mixed cell distribution pattern of one cell population in relation to another cell population. **(B)** Unmixed point pattern distribution of cell phenotype A and B. **(D)** Graphic representation of the image shown in B using the cross-G-function, showing the increased distance of the G(*r*) curve from the theoretical estimate curve (Poisson curve), characterizing a clustering or unmixed cell distribution pattern of one cell population in relation to another cell population. The graphics were generated using R studio software version 3.6.1.

The most common way to study the random process of cell placement, given certain intensity patterns of spatial distribution between two groups of cell phenotypes (*i*-to-*j*), is to compare the theoretical curve with the empirical nearest neighbor cross-G-function, *G*_*i,j*_(*r*) = *P*{*d*(*u,X_j*)|*u* ∈*X*_*i*_} (Baddeley and Turner, 2005). Overall, there are theoretically three possible patterns of distribution when the empirical curve is above, close, or below the theoretical curve: regular, random, and cluster, respectively. However, the regular pattern does not tend to occur in nature, and hence a situation in which the empirical curve is very far above the theoretical curve should be used with caution. Empirical curves that occur only slightly above the theoretical curve are more accurately interpreted as close to a random pattern than as a potential regular pattern. When studying the distribution of two different cell phenotypes, such as cell phenotype A (malignant cells) and cell phenotype B (lymphocytes), a researcher should typically recognize only two patterns—random or mixed (when the empirical curve is close to the theoretical curve, either above or below) and cluster or unmixed (when the empirical curve is below the theoretical curve)—related to cell phenotype A. These two patterns of distribution can be represented graphically (Figure 9). Specifically, when the empirical cross-G-function is plotted against the theoretical expectation or Poisson curve, the shape of the function indicates how the events are spaced in a point pattern of two cell phenotypes. If the events of cellular distribution are random or mixed (e.g., cell phenotype B and cell phenotype A are mixed together in the plot, Figures 9A,C), then the nearest neighbor cross-G-function is very close to the Poisson curve because the probability of a neighbor being close is high. In contrast, as the distance increases between the empirical cross-G-function and the Poisson curve, the events are more spaced and a cluster or unmixed pattern can be identified in the plot, as shown in Figures 9B,D, where cell phenotype B is in separate clusters from cell phenotype A. To determine the probability that cell phenotypes have a random or cluster pattern related to the theoretical curve, the researcher must process several images from the project to ensure that a clear threshold is present to eliminate the possibility of a random pattern (Parra et al., 2021).

## Correlation Functions

**FIGURE 10:**
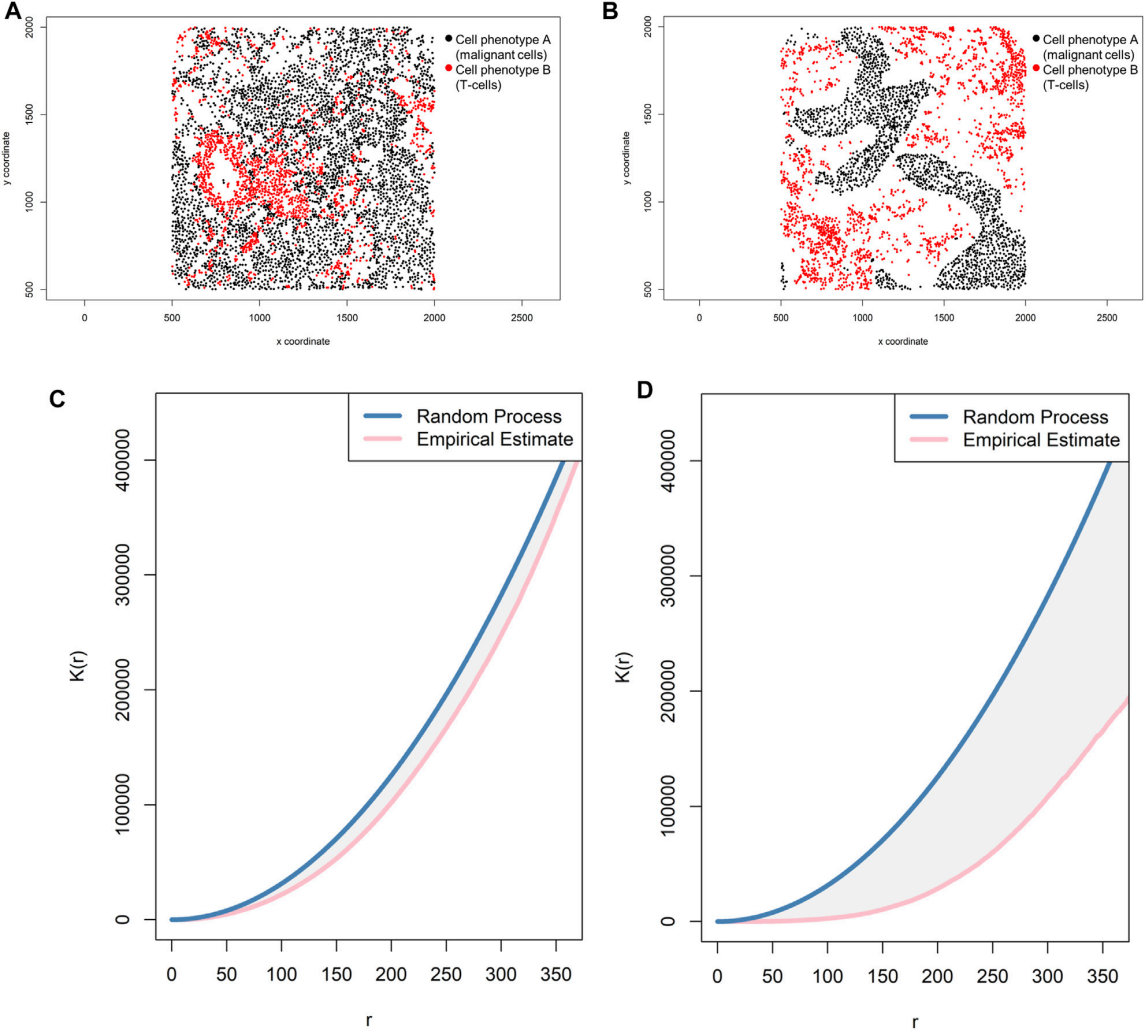
Representative graphs showing the K-function [K(*r*)] curve of cellular distributions between two groups of cells from a representative point pattern of multiplex immunofluorescence images of lung adenocarcinoma. **(A)** Random point pattern distribution of cell phenotype A and B. **(C)** K(*r*) curve extracted from the image shown in A, showing the proximity of the curve to the theoretical estimate curve (Poisson curve), characterizing a random or mixed distribution pattern of the two cell phenotypes. **(B)** Unmixed point pattern distribution of cell phenotype A and B. **(D)** K(*r*) curve extracted from the image shown in B, showing the increased distance of the curve from the theoretical estimate curve (Poisson curve), characterizing a clustering or unmixed distribution pattern of cell phenotype A and B. The graphics were generated using R studio software version 3.6.1.

Correlation functions basically provide information about how many specific cells of a certain phenotype (e.g., intensity of cell phenotype B) are within a certain radius (*r*) from another cell phenotype (e.g., cell phenotype A) and can give a good sense of the different levels of interaction between two cell populations in terms of point intensity level (λ) or number of cells (Figure 8B). A commonly used correlation function for spatial analysis is the K-function: *K*_*i,j*_(*r*)=(*E*{*n*[*X*_j_∩*b*(*u*,*r*)]|*u*∈*X*_i_})/*λ*_*j*_ (Baddeley and Turner, 2005; Lagache et al., 2013). The K-function essentially normalizes the spatial distribution from one cell phenotype to another cell phenotype by the intensity of the cells present in the radius. As in the cross-G-function, to determine if cell phenotype B has a distinct pattern of distribution related to cell phenotype A, one can calculate the theoretical correlation function for a random process using the same principle, and observed graphical changes can indicate that cells of phenotype B are displaced in random patterns (Figures 10A,C) or cluster patterns related to cells of phenotype A (Figures 10B,D). This function determines the consistency of the observed distribution of distances among all cells located in spatial images, using the theoretical distribution for the Poisson model as a benchmark.

## Complementary Functions

Thus far, I have described two spatial functions, the G- and K-functions, which are the most common functions used for spatial image analysis. These two functions combined can provide valuable characterization of the distribution of different cell types in an image. In the learning theory literature, this is known as feature construction or extraction. The G-function provides information about the distribution of the closest cells to another cell type, and the K-function provides the context for the density of these neighbors. In some scenarios, the G-function demonstrates that cells of phenotype B are likely to be within a certain radius of cells of phenotype A, but the K-function demonstrates the intensity of the cell phenotype B distribution from cell phenotype A at the same ratio. Combined, these two distance functions can generate a compressive analysis about the tumor microenvironment, characterizing the proximity and level of interaction between one cell phenotype and another (Parra et al., 2021; Parra, Ferrufino-Schmidt, et al., 2021).

Understanding of the data provided by these two basic functions, in terms of spatial analysis of cell distribution in an image, may be improved by using a complementary function. Complementary functions are derived from the cross-G- and K-function to provide more information about cell distribution patterns and correct transformation that can occur in the image to better reflect the features observed visually.

**FIGURE 11:**
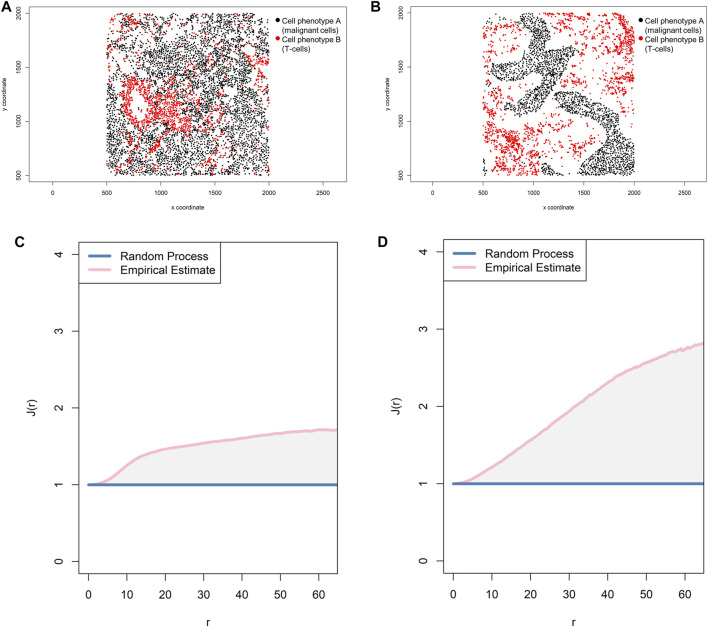
Representative graphs showing the J-function [J(*r*)] for two different cell populations, illustrating the cellular distribution between the two groups of cells using a representative point pattern in multiplex immunofluorescence images of lung adenocarcinoma. **(A)** Random point pattern distribution of cell phenotype A and B. **(C)** Graphic representation of the J(*r*) curve showing its lineup with the theoretical estimate (Poisson line), characterizing a random or mixed pattern of one cell population in relation to another cell population. **(B)** Unmixed point pattern distribution of cell phenotype A and B. **(D)** Graphic representation of the J(*r*) curve showing highly increased distance above the theoretical estimate (Poisson line), characterizing a clustering or unmixed pattern of one cell population in relation to another cell population. The graphics were generated using R studio software version 3.6.1.

One transformation correction that can be incorporated into the basic functions described above is the J-function: *J*_*i,j*_(*r*) *=* [1 − *G*_*i,j*_(*r*)]*/*[1 *F*_*j*_(*r*)] (Baddeley and Turner, 2005). This function is used to compare distances from an arbitrary point to the nearest neighbor (empty-space F-function: [*F*_*j*_(*r*) = *P*{*d*(*u,X*_j_)≤*r*}]) (Baddeley and Turner, 2005) and distances from a typical point in the pattern measured using the nearest neighbor distance cross-G-function (Figure 8C). If the distance in the J-function distribution follows the Poisson process, deviation of the J-function by more than 1 indicates spatial randomness and deviation by less than 1 indicates clustering (Figure 11). One can then estimate the empty-space F-function, which is identical to the G-function when the pattern is random but different from it when the probability of not observing another cell fluctuates (Kather et al., 2015; Zheng et al., 2020). Hence, this J-function aids in identifying any pockets of empty space around cells.

**FIGURE 12:**
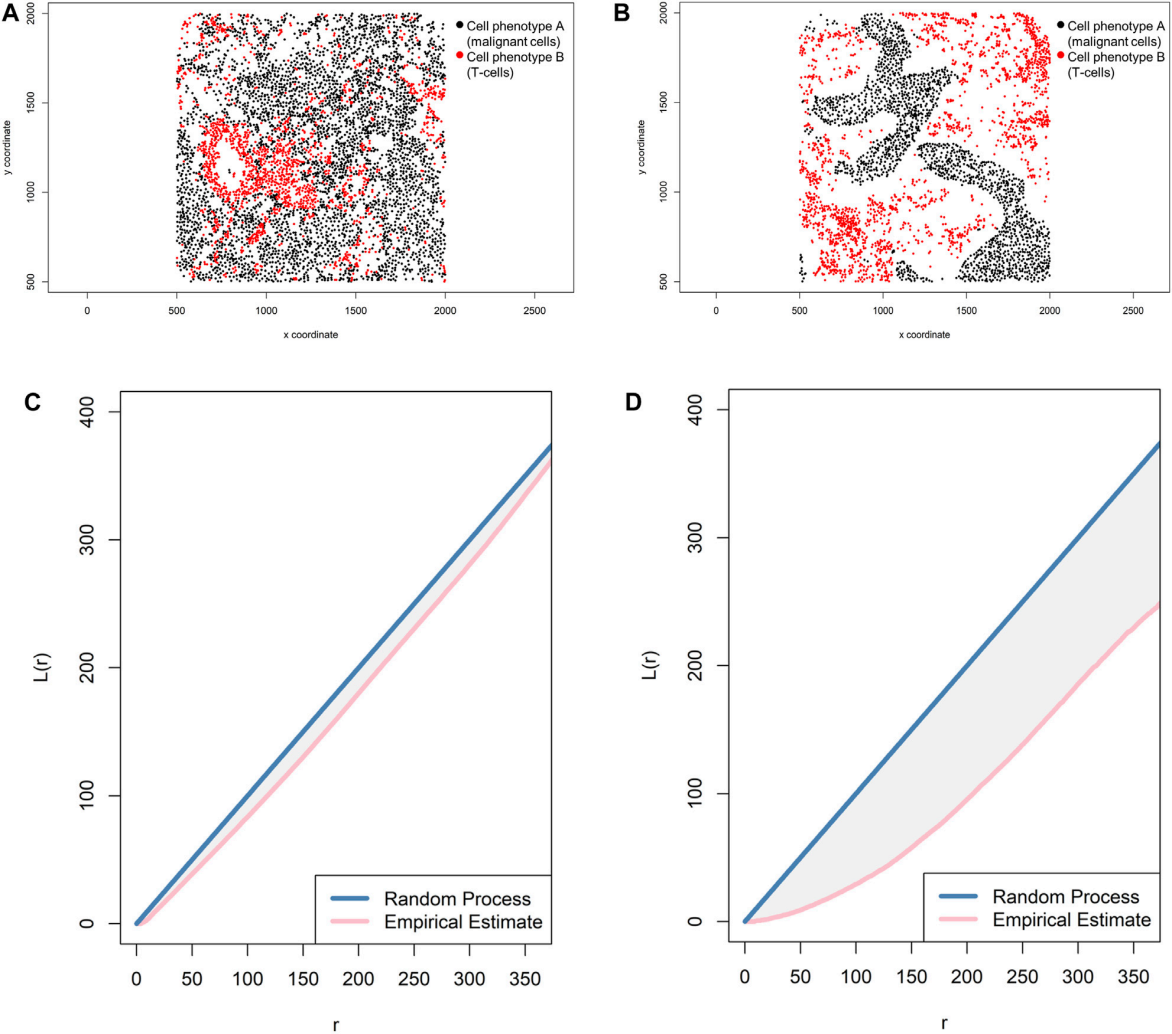
Representative graphs showing the L-function [L(*r*)] for two different cellular distribution patterns between two group of cells from a representative point pattern in multiplex immunofluorescence images of lung adenocarcinoma. **(A)** Random point pattern distribution of cell phenotype A and B. **(C)** Graphic representation of the L(*r*) line showing its proximity to the theoretical estimate (Poisson line), characterizing a random or mixed pattern of one cell phenotype in relation to another cell phenotype. **(B)** Unmixed point pattern distribution of cell phenotype A and B. **(D)** Graphic representation of the L(*r*) line showing that it is located far from the theoretical estimate (Poisson line), characterizing a clustering or unmixed pattern of one cell phenotype in relation to another cell phenotype. The graphics were generated using R studio software version 3.6.1.

In addition, the L-function—L_*i,j*_(*r*) = √[(*K*_*i,j*_(*r*)]/π) (Baddeley and Turner, 2005)—can complement a spatial imaging study. Mathematically, this function is simply the square root of K-function divided by pi, and it helps visualize the K-function as a linear shape when it is graphically represented and can identify small differences in cell pattern distributions that are sometimes difficult to identify with the K-function. When the L-function is represented graphically, one should observe a seemingly straight line whenever the pattern is random (Figure 12).

**FIGURE 13:**
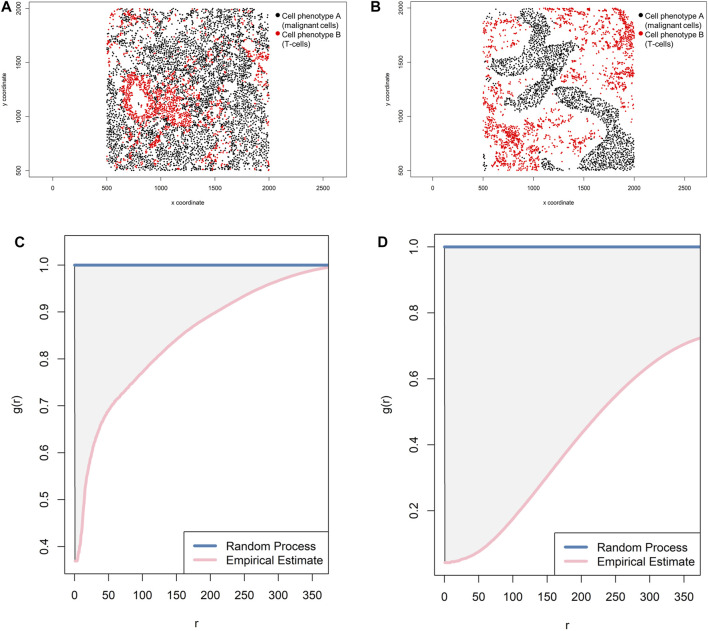
Representative graphs showing the pair correlation function [g(*r*)] for two different cellular distribution patterns between two group of cells from a representative point pattern in multiplex immunofluorescence images of lung adenocarcinoma. **(A)** Random point pattern distribution of cell phenotype A and B. **(C)** Graphic representation of the g(*r*) curve showing a line forming downward from the theoretical estimate (Poisson line), characterizing a random or mixed pattern of one cell phenotype in relation to another cell phenotype. **(B)** Unmixed point pattern distribution of cell phenotype A and B. **(D)** Graphic representation of the g(*r*) curve showing increased distance below the theoretical estimate (Poisson line), characterizing a clustering or unmixed pattern of one cell phenotype in relation to another cell phenotype. The graphics were generated using R studio software version 3.6.1.

Lastly, the pair correlation function—*g*_i,j_(*r*) = [*K*_*i,j*_'(*r*)]/2π*r* (Baddeley and Turner, 2005)—is easy to understand but more complicated to estimate than the other functions (Gavagnin et al., 2018). The pair correlation function is related to the K- and L-functions; it is a modified version of the K-function where instead of summing all points (cell phenotypes) within a given radius, points falling within a narrow distance band are summed, and the result is the dependence between two different points or two different cell populations. If the *g*(*r*) is more than 1, then the points or the correlation between the two cell groups at or around a certain radius are more clustered and the *g* curve is far below the Poisson curve process. If the *g*(*r*) is less than 1, then the points or the correlation between the two cell groups are more dispersed and the *g* curve is just below the Poisson curve process (Figure 13). The *g*(*r*) can never be less than 0.

## Statistical Analysis Modeling

As with any other statistical analysis, the data obtained from spatial analysis can be used to perform univariate or multivariate analysis with several metrics, and data may be associated with clinicopathologic information in some meaningful way. A simple mathematical model can be applied to investigate the effect of different patterns of distribution for different cell phenotypes in the images on clinical information. Researchers would like to determine if the spatial distribution of certain cell phenotypes can be influenced by the type of tumor and, moreover, as the ultimate goal, if the cellular distribution pattern can predict response to treatment. Several statistical methods, including some of the more common methods such as generalized linear models, form the basis of most supervised machine learning methods, nonparametric testing, clustering methods, Bayesian methods, penalized regression models, survival analysis, dimensionality reduction, and others that can be applied to interpret the data (Baddeley and Turner, 2005; Illian et al., 2008; Demidenko 2020).

### Cluster Analysis Methods

To characterize the tumor microenvironment data obtained from mIF imaging, researchers must identify different cell subpopulations, and this can be achieved via cluster analysis. Although cluster methods are not a measurement of distance and are not frequently used to interpret the type of data presented in this paper, cluster methods can be used for exploratory analysis of the data, in which observations are divided into different groups with standard features to ensure that the groups meaningfully differ as much as possible.

The two main types of classification are K-means clustering and hierarchical clustering. K-means clustering can be used when the number of classes is fixed; this method is infrequently used in mIF data. In contrast, hierarchical clustering can be used for an unknown number of classes and is probably more appropriate for classifying cell phenotypes.

**FIGURE 14:**
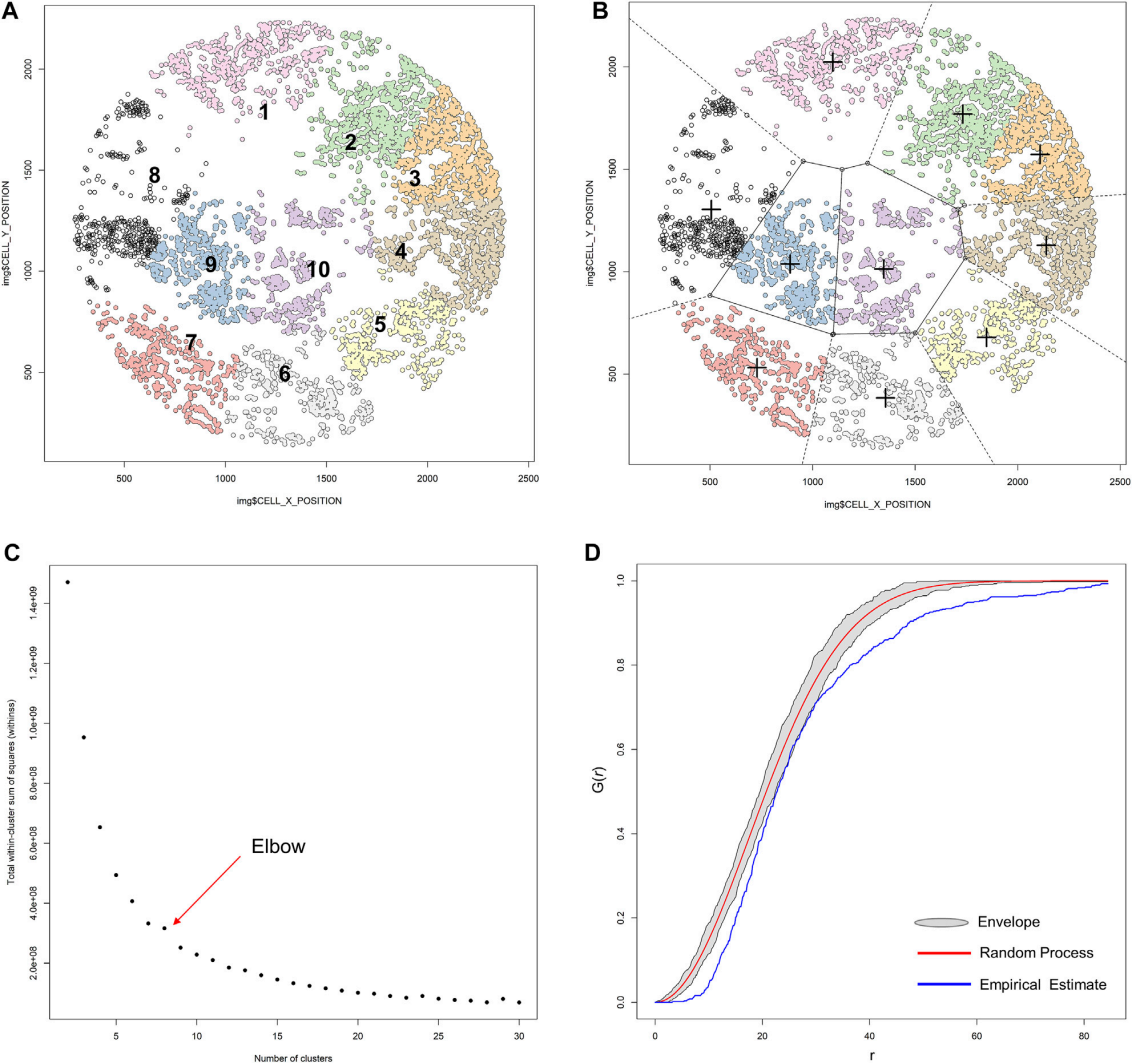
K-means unsupervized clustering, elbow, and envelope applied in multiplex immunofluorescence image data. **(A)** K-means unsupervized clustering showing ten groups (represented by the colored points) of non-overlapping clusters in which the inner points of the cluster are as similar as possible within the image. **(B)** Different clusters are maintained in different spaces, and the center (**+**) of each cluster is located such that it is the arithmetic mean of the data points in the cluster. **(C)** Graphic representation of the elbow method, showing the sum of squares and the number of clusters plotted into a curve. The point of the elbow in the curve indicates the optimum number of clusters. **(D)** Graphic representation of an envelope to estimate the variation of data to achieve efficiency gains, showing the minimal variation of the envelope related to the random process curve from a representative sample of multiplex immunofluorescence data points. The graphics were generated using R studio software version 3.6.1.

K-means clustering comprises unsupervized learning methods of vector quantization that have an iterative process in which data are grouped into *k* predefined non-overlapping clusters or subgroups, making the inner points of the cluster as similar as possible (Figure 14A). To maintain different clusters in distinct spaces, K-means clustering allocates the data points to a cluster in such a way that each observation belongs to the cluster with the nearest mean (cluster center or centroid), so that the sum of the squared distance between the cluster centroid and the data point is minimized; at this position, the centroid of the cluster is the arithmetic mean of the data points that are in the clusters (Figure 14B). This results in a partitioning of the data space into Voronoi cells (Schuffler et al., 2015). Less variation in the cluster results in similar or homogeneous data points within the cluster.

To identify the number of clusters in determinate data, we use the elbow or the purpose method. In the elbow method, the sum of squares and the number of clusters are plotted into a curve resembling a human elbow; the point of the elbow in the curve indicates the optimum number of clusters and the point after the elbow point indicates the final value of the number of clusters (Figure 14C).

**FIGURE 15:**
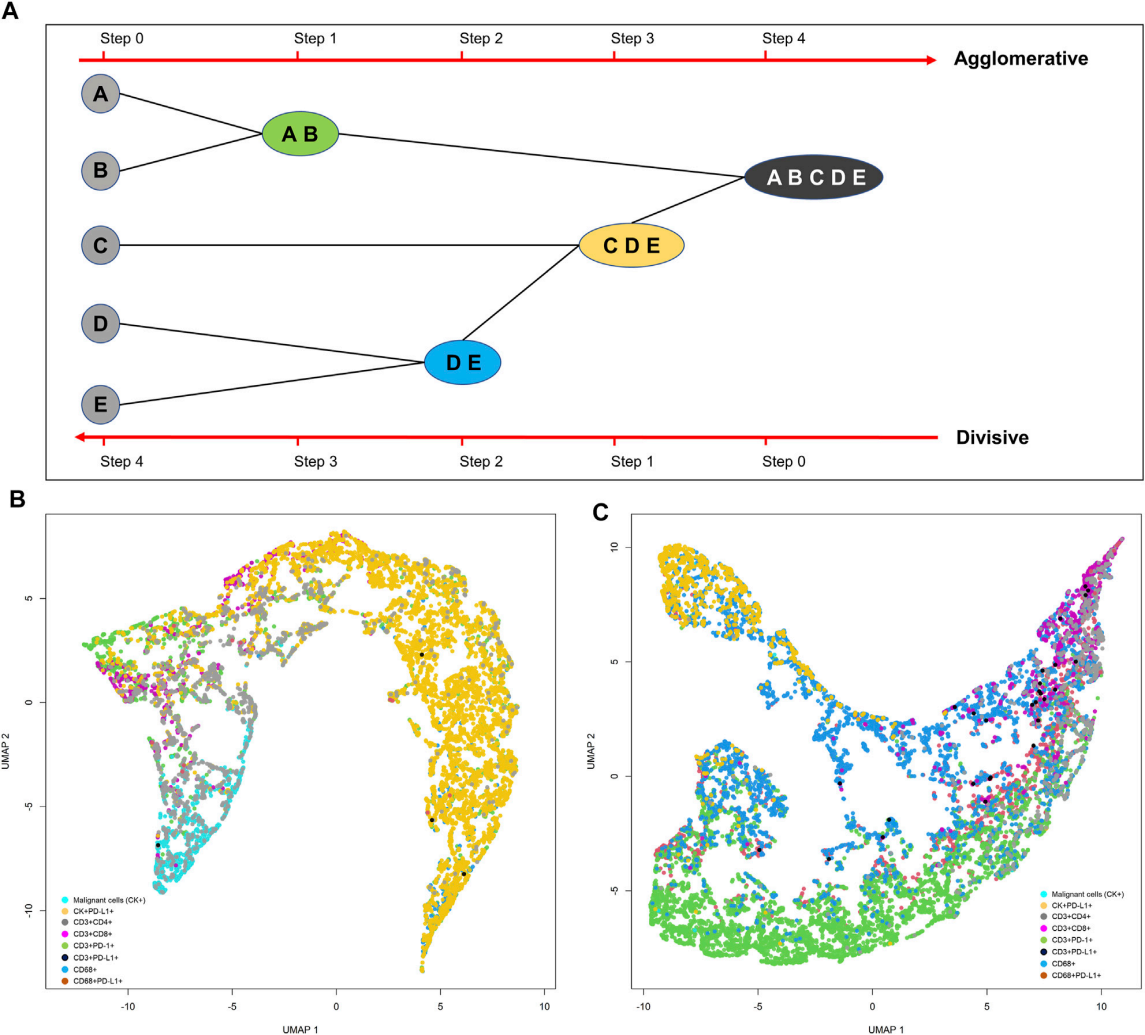
Representative schema of hierarchical and divisive clustering to agglomerate similar objects into groups **(A)**. Uniform manifold approximation and projection (UMAP) showing different cell population distribution patterns extracted from two different lung adenocarcinoma cases analyzed with multiplex immunofluorescence against malignant cells [cytokeratin (CK)+] and CD3+, CD4+, CD8+, PD-1+, PD-L1+, and CD68+ antibodies **(B, C)**. The graphics B and C were generated using R studio software version 3.6.1.

Although the K-means clustering algorithm can be used in image segmentation, image compression, vector quantization, clustering analysis, machine learning, and other methods, the algorithm requires prior specification of the number of cluster centers, and if there are overlapping data the algorithm cannot distinguish clusters very well. Depending on how the data are presented, the results generated can be different every time the algorithm is run, and the Euclidean distance can unequally weight factors and can be used only if the meaning is defined. In contrast, hierarchical clustering can be agglomerative when similar objects are grouped into clusters and into a set of clusters, where each cluster is distinct from the others and the objects within each cluster are broadly similar to each other (Comin et al., 2014; Lin et al., 2015) (Figure 15A). Divisive hierarchical clustering is done by initially grouping all observations into one cluster and then successively splitting these clusters, typically by sequentially merging similar clusters (Figure 15A). The similarity here is the distance among points, which can be computed in many ways, and this distance is the crucial element of discrimination. However, in practice, divisive hierarchical clustering is rarely done. Unfortunately, it is not possible to undo the previous steps after applying the algorithm, and when the clusters have been assigned, they can no longer be moved around. In addition, this method is not suitable for large datasets, the order of the data affects the results, and the method is very sensitive to data outliers.

With any data, the efficiency of multivariate parameter estimation and prediction must be increased by exploring variation of the data, which is done using envelope methods. Envelopes achieve efficiency gains by basing estimation on the variation of the data. The Monte Carlo method (Figure 14D) is a type of computational envelope algorithm that uses the random repletion of the sampling to obtain numeric results that optimize, integrate, and generate draws from a probability distribution of the data (Sanchez et al., 2021). Monte Carlo tests are related to the randomization tests commonly used in nonparametric statistics.

### Dimensional Reduction Methods for Data Visualization

Because we generate highly multiparametric single-cell data using mIF, statistical methods can be used for better visualization and dimensional reduction, providing a location for each data point on a two- or three-dimensional map. This type of visualization through dimensional reduction algorithms tends to fall into one of two overall categories, algorithms that seek to preserve the distance structure within the data and algorithms that favor the preservation of local distances over global distance; these algorithms are applied for cell phenotype data visualization. Algorithms such as principal component analysis (PCA), multidimensional scaling, and Sammon mapping fall into the first category, and t-distributed stochastic neighbor embedding (t-SNE) and uniform manifold approximation and projection (UMAP), as well as others, fall into the second category (Tsogo et al., 2000).

PCA is an unsupervized algorithm that can create linear combinations of the original features, and then the new features are orthogonal, which means that they are uncorrelated (Rohde, 2002). Because the reduction of the data is dependent on scale, the dataset must be normalized before this technique can be performed (Rohde, 2002). Several algorithm variations, such as kernel PCA or sparse PCA, can be applied to compare the performance of the data, but an important disadvantage is the necessity of manually setting or tuning the threshold for cumulative explained variance.

Multidimensional scaling is another reduction method frequently used to translate information about pairwise distances obtained from data among a set number of points mapped into an abstract Cartesian space (Jackle et al., 2016). This method allows construction of a distance matrix with the distances between each pair of objects in a set placing each object into a dimensional space, providing a point pattern to be visualized on a scatter plot.

Sammon mapping is another algorithm used in exploratory analysis. This method translates a map with a high-dimensional space to a space of lower dimensionality by trying to preserve the structure of inter-point distances from the high-dimensional space in the lower-dimension projection. Sammon mapping is considered a nonlinear approach because the mapping cannot be represented as a linear combination of the original variables, as is possible in techniques such as PCA, and this also makes Sammon mapping more difficult to use for classification applications.

For high-dimensional data such as that obtained by image analysis, a reduction and visualization can be made through t-SNE or UMAP reduction analysis (Wu et al., 2019). t-SNE is a statistical method for visualizing high-dimensional data by giving each data point a location in a two- or three-dimensional map. It is based on SNE, originally developed by Sam Roweis and Geoffrey Hinton (Van der Maaten and Rey Hinton, 2008). t-SNE constructs a probability distribution over pairs of high-dimensional objects in such a way that similar objects are assigned a higher probability while dissimilar points are assigned a lower probability using the Euclidian distance between objects (Figures 15C,D). The visual clusters often require good understanding because they can be influenced by the parameterization, forcing exploration of different parameters to validate the results. Although t-SNE is incredibly flexible and can often find structure where other dimensionality-reduction algorithms cannot, that very flexibility makes t-SNE tricky to interpret.

UMAP is another dimension reduction technique that can be used for data visualization similar to that described for t-SNE, but UMAP can be applied for general nonlinear dimension reduction (Becht et al., 2018). UMAP is based on distances between the observations obtained by the data rather than the source features, and it does not have an equivalent of the factor loadings that are required for linear techniques such as PCA. Importantly, as a way to improve the computational efficiency of the UMAP algorithm, several approximations can be made and small data sizes (less than 500 samples) can be analyzed (Wu et al., 2019).

In summary, spatial distance analysis methods can be applied to analyze the spatial distribution of cells determined by mIF data. There are several methods to analyze the distribution of different cell phenotypes, but the most simple approach is a combination of cell phenotype compartmentalization at a tissue level with nearest neighbor distance measurement through the cross-G- and K-function at a cellular level to identify patterns of distribution and interaction between cell phenotypes. Although cluster analysis and visualization methods are important in exploring mIF data, overall no single cluster or visualization method described here outperforms another in terms of identifying the characteristics of the data, and for this reason researchers can choose the most convenient method for interpreting their results. Given this situation, approaches for cellular cluster identification should allow subsequent in-depth analysis to identify new clusters of special cell phenotypes and permit interpretation of features that contribute to the analysis, thus effectively answering the research question or providing a potential clinical application.
